# Supplementary material for: Examining Facilitators of HPV Vaccination Uptake in Men Who Have Sex with Men: A Cross-Sectional Survey Design
Source: Int J Environ Res Public Health. 2020 Oct 22;17(21):7713. doi: 10.3390/ijerph17217713 (PMC7672608; doi:10.3390/ijerph17217713)
Supplement: Supplementary file 1 [file ijerph-17-07713-s001.zip › ijerph-958272-supplementary.docx]

Supplementary Materials

**Table S1.** The questionnaire used in the present research.

Note: section headings (blue) were not shown to participants.

| **Item number** | **Construct** | **Item**  **(Source)** | **Response scale** |
| --- | --- | --- | --- |
| **Screening questions** | | | |
| Q1 | Location | Where do you live? | 1. England 2. Wales 3. Scotland 4. Northern Ireland 5. Other (outside the United Kingdom) |
| Q2 | Gender | What is your gender? | 1. Male / male-presenting 2. Female / female-presenting 3. Gender non-conforming 4. Other   *Phrasing for survey advertised by Terrence Higgins Trust:*   1. *Male (including trans men)* 2. *Female (including trans women)* 3. *Gender non-conforming* 4. *Other* |
| Q3 | Sexual orientation | Which of the following most closely matches your sexual orientation? | 1. Gay / homosexual / only attracted to own gender (men) 2. Bisexual / pansexual / attracted to own gender (men) 3. Heterosexual / straight / only attracted to women 4. Something else 5. Not sure |
| Q4 | Age | What is your age? |  |
| *If [Q1 = 2, 3, 4, 5] or [Q2 = 2, 3, 4] or [Q4 < 18 or > 45], participant was redirected to ineligibility page.*  *Ineligibility message read: “Thank you for your interest in our research. Unfortunately, our study criteria are living in England, being a man who has sex with men, and being age 18-45. You do not appear to meet our study criteria. If you believe you do meet the criteria, please click here to go back and change your answers appropriately. Have a nice day!”* | | | |
| **Disease perceptions** | | | |
| Q5 | Awareness of HPV (1) | Have you heard of human papillomavirus (HPV) before today?  [1] | 1. Yes 2. No 3. I don’t know |
| Q6 | Awareness of HPV (2) | How much would you say you know about HPV? | 1. None at all 2. A little 3. A moderate amount 4. Quite a lot |
| Q7 | Perceived severity of HPV  *Health Belief Model (HBM) construct* | How serious would it be for you if you became infected with HPV?  [2] | 1. Not at all serious 2. Somewhat serious 3. Mildly serious 4. Very serious 5. Extremely serious |
| Q8 | Awareness of anal cancer | How much would you say you know about anal cancer?  [1] | 1. None at all 2. A little 3. A moderate amount 4. Quite a lot |
| Q9 | Perceived severity of anal cancer  *HBM construct* | How much do you think having anal cancer would affect your life?  [1] | 1. Not at all 2. A little 3. A moderate amount 4. Quite a lot |
| **Knowledge** | | | |
|  | Knowledge introduction | Please look at these statements and say whether you think each one is true or false. If you haven’t heard of HPV, you may select 'I don't know'. | |
| Q10 | Knowledge | You can get HPV from having sex.  [1] | 1. True 2. False 3. I don’t know |
| Q11 | Knowledge | HPV infection is rare.  [1] | 1. True 2. False 3. I don’t know |
| Q12 | Knowledge | Only women can get HPV. | 1. True 2. False 3. I don’t know |
| Q13 | Knowledge | HPV can cause cervical cancer. | 1. True 2. False 3. I don’t know |
| Q14 | Knowledge | HPV can cause genital herpes.  [1] | 1. True 2. False 3. I don’t know |
| Q15 | Knowledge | HPV can cause anal cancer.  [1] | 1. True 2. False 3. I don’t know |
| Q16 | Knowledge | Only people who have sex with lots of other people get HPV.  [1] | 1. True 2. False 3. I don’t know |
|  | HPV statement | HPV is a common sexually transmitted disease (STD) that can cause genital warts and cervical cancer. HPV does not cause genital herpes.  Men who have sex with men (MSM) are at risk of other cancers linked to HPV, such as cancer of the anus, penis, mouth or throat.  MSM are also at risk of genital warts caused by types of HPV that do not cause cancer.  [1] | |
| **Vaccine perceptions** | | | |
|  | Vaccine statement | The next questions are about the HPV vaccine, sometimes called Gardasil. You may have seen information promoting the vaccine for preventing genital warts and cervical cancer in girls and young women: The first dose of the HPV vaccine is routinely offered to girls in school year 8.  (Adapted from 1) | |
| Q17 | Awareness of vaccine  *Categorization by PAPM* | Have you ever heard of the HPV vaccine before today?  [1] | 1. Yes 2. No 3. I don’t know |
| Q18 | Female-only belief | ‘I thought that only women and girls are supposed to get the HPV vaccine.’ | 1. Strongly agree 2. Somewhat agree 3. Neither agree nor disagree 4. Somewhat disagree 5. Strongly disagree |
|  | Vaccine statement | Gay and bisexual men up to the age of 45 are eligible for free HPV vaccination on the NHS when they visit sexual health clinics and HIV clinics in England. More information is available at the end of this questionnaire. | |
| Q19 | Information sources | Have you ever heard about the HPV vaccine from any of these sources? Check all that apply.  If you have not heard about the HPV vaccine, please leave this question blank.  [1] | 1. Doctor or health care provider 2. Friend or family member 3. Brochure or poster 4. Commercial or ad from a drug company 5. Other [blank space] |
| Q20 | Perceived risk of HPV  *HBM construct* | If someone doesn't get vaccinated for HPV, how likely is it that they will become infected with HPV at some point in their life?  [2] | 1. Very unlikely 2. Unlikely 3. Neither likely nor unlikely 4. Likely 5. Very likely 6. I don’t know |
| Q21 | Attitude to vaccine  *Theory of Planned Behaviour (TPB) construct* | Is your opinion of the HPV vaccine…  [1] | 1. Mostly positive 2. Somewhat positive 3. Neutral / no opinion 4. Somewhat negative 5. Mostly negative |
| Q22 | Perceived benefits  *HBM construct* | ‘Getting the HPV vaccine is a good thing for someone like me to do for their overall health.’  (Adapted from 2) | 1. Strongly agree 2. Somewhat agree 3. Neither agree nor disagree 4. Somewhat disagree 5. Strongly disagree |
| Q23 | Self-efficacy  *HBM construct* | 'I feel confident in my ability to get vaccines (such as the HPV vaccine) from a sexual health clinic.’  (Adapted from 2) | 1. Strongly agree 2. Somewhat agree 3. Neither agree nor disagree 4. Somewhat disagree 5. Strongly disagree |
| Q24 | Subjective norms  *TPB construct* | ‘Most people who are important to me would want me to get the HPV vaccine.'  (Adapted from 2) | 1. Strongly agree 2. Somewhat agree 3. Neither agree nor disagree 4. Somewhat disagree 5. Strongly disagree |
| Q25 | Anticipated regret | Imagine that you did not get the HPV vaccine and you got an HPV infection that could lead to anal cancer. The HPV vaccine might have prevented this. How much would you regret that you did NOT get the HPV vaccine?  (Adapted from 1) | 1. Not at all 2. A little 3. A moderate amount 4. Quite a lot |
|  |  | For the next questions, if you are unsure, please tell us what you think the answer might be.  [1] | |
| Q26 | Perceived effectiveness (HPV) | How much protection does the HPV vaccine give against developing HPV?  [1] | 1. No protection 2. A little protection 3. A moderate amount of protection 4. Quite a lot of protection 5. Complete protection |
| Q27 | Perceived effectiveness (anal cancer) | How much protection does the HPV vaccine give against anal cancer?  [1] | 1. No protection 2. A little protection 3. A moderate amount of protection 4. Quite a lot of protection 5. Complete protection |
| **PAPM categorization** | | | |
| Q28 | Clinic attendance  *Categorization by PAPM* | Have you attended a sexual health or HIV clinic since summer 2016? | 1. Yes 2. No 3. Uncertain |
| *If [Q28 = 2], participant was not given Q29 and Q30.* | | | |
| Q29 | Sexual orientation disclosure | At your visit to the sexual health or HIV clinic, did you tell the doctor/nurse about your sexuality or that you have had sex with a man previously? | 1. Yes 2. No 3. Uncertain |
| Q30 | HCP recommendation  *Categorization by PAPM* | At your visit to the sexual health or HIV clinic, were you offered the HPV vaccine (Gardasil)? | 1. Yes 2. No 3. Uncertain |
| Q31 | Willingness to disclose | 'I am willing to tell a doctor or nurse my sexual orientation / the fact that I have sex with men.’ | 1. Strongly agree 2. Somewhat agree 3. Neither agree nor disagree 4. Somewhat disagree 5. Strongly disagree |
| Q32 | Vaccination status  *Categorization by PAPM* | Which of the following best describes you? | 1. I am undecided about having the HPV vaccine. 2. I have decided not to have the HPV vaccine. 3. I have decided to have the HPV vaccine, but I have not yet had any injections. 4. I have decided to have the HPV vaccine, and I have had at least one injection. 5. I have decided to have the HPV vaccine, but I am not sure if I have had any injections. |
| *Only if [Q32 = 4], participant was shown Q33.* | | | |
| Q33 | Vaccination status  *Categorization by PAPM* | How many of the HPV vaccine injections have you had?  [3] | 1. I’ve had 1 injection. 2. I’ve had 2 injections. 3. I’ve had 3 injections. 4. Uncertain. |
| **Potential barriers** | | | |
| Q34 | Perceived behavioural control  *TPB construct* | ‘There are factors beyond my control that can prevent me from getting vaccines.’  (Adapted from 2) | 1. Strongly agree 2. Somewhat agree 3. Neither agree nor disagree 4. Somewhat disagree 5. Strongly disagree |
| Q35 | Fear of side effects (short term) | ‘I am concerned that vaccines (such as the HPV vaccine) could cause short term health problems, like pain or discomfort.’  (Adapted from 1) | 1. Strongly agree 2. Somewhat agree 3. Neither agree nor disagree 4. Somewhat disagree 5. Strongly disagree |
| Q36 | Fear of side effects (long term) | ‘I am concerned that vaccines (such as the HPV vaccine) could cause lasting health problems.’  (Adapted from 1) | 1. Strongly agree 2. Somewhat agree 3. Neither agree nor disagree 4. Somewhat disagree 5. Strongly disagree |
| Q37 | Logistical barriers | ‘I am generally too busy to get vaccines.' | 1. Strongly agree 2. Somewhat agree 3. Neither agree nor disagree 4. Somewhat disagree 5. Strongly disagree |
| Q38 | Medication interaction | ‘I am concerned the HPV vaccine could interact with other medications (e.g. HIV medication).' | 1. Strongly agree 2. Somewhat agree 3. Neither agree nor disagree 4. Somewhat disagree 5. Strongly disagree |
| Q39 | Need for multiple shots | ‘It is a hassle to get all three HPV vaccine injections.’ | 1. Strongly agree 2. Somewhat agree 3. Neither agree nor disagree 4. Somewhat disagree 5. Strongly disagree |
| **Demographic information** | | | |
| Q40 | Ethnicity | What is your ethnic group?  [4] | 1. White: English/ Welsh/ Scottish/ Northern Irish/ British 2. White: Irish 3. White: Gypsy or Irish Traveler 4. Any other white background 5. Mixed/multiple: White and Black Caribbean 6. Mixed/multiple: White and Black African 7. Mixed/multiple: White and Asian 8. Any other Mixed/ multiple ethnic background 9. Asian/Asian British: Indian 10. Asian/Asian British: Pakistani 11. Asian/Asian British: Bangladeshi 12. Asian/Asian British: Chinese 13. Any other Asian background 14. Black: African 15. Black: Caribbean 16. Any other Black/ African/ Caribbean background 17. Other ethnic group: Arab 18. Any other ethnic group 19. Prefer not to say 20. Other (please write) |
| Q41 | Education | Please select the highest level of education you have completed. | 1. Primary school 2. Secondary school 3. Trade/technical school 4. Bachelor’s degree 5. Post-graduate study |
| Q42 | Hepatitis B vaccination | The hepatitis B vaccine is a series of three injections. How many hepatitis B vaccine injections, if any, have you had? | 1. 0 2. 1 3. 2 4. 3 5. At least one shot, but don’t know how many 6. I don’t know if I have had any shots |
| Q43 | Number of sexual partners | In your lifetime, with how many different people have you had vaginal, anal, or oral sex? This question is optional.  [1] |  |
| **Post-questionnaire information** | | | |
|  | Post-questionnaire information | Thank you very much for participating in our research! Here is some further information about the topics covered in this survey:  Men who have sex with men (MSM) up to and including the age of 45 are eligible for free HPV vaccination on the NHS when they visit sexual health or HIV clinics. This new programme has been gradually rolling out across GUM and HIV clinics in England from April 2018.  For more information about how to get the vaccine, click here [5] or here [6] (these links will open in a new tab).  After receiving the HPV vaccine, it is very common to experience short-term headaches and pain at the injection site. It is common to experience a high temperature, nausea, or bruising or itching at the injection site. For more information on side effects, click here [7] (this will open in a new tab).  People being treated with high doses of corticosteroids, other immunosuppresants, chemotherapy, or radiotherapy should talk to their doctors about when / how it is best to get the vaccine. For more information click here (8) (this will open in a new tab). | |

Questionnaire references

1 Reiter, L.P.; Brewer, T.N.; McRee, S.A.–L.; Gilbert, B.; Smith, J. Acceptability of HPV Vaccine Among a National Sample of Gay and Bisexual Men. *Sex. Transm. Dis.* **2010**, 37, 197–203, doi:10.1097/OLQ.0b013e3181bf542c.

2 Gerend, A.M.; Madkins, A.K.; Phillips, A.G.; Mustanski, B. Predictors of Human Papillomavirus Vaccination Among Young Men Who Have Sex With Men. *Sex. Transm. Dis.* **2016**, 43, 185–191, doi:10.1097/OLQ.0000000000000408.

3 Barnard, M.; George, P.; Perryman, M.L.; Wolff, L.A. Human papillomavirus (HPV) vaccine knowledge, attitudes, and uptake in college students: Implications from the Precaution Adoption Process Model. *PLoS ONE* **2017,** 12, e0182266, doi:10.1371/journal.pone.0182266.

4 Household Questionnaire: England. 2011. Available online: [www.census.gov.uk](http://www.census.gov.uk). [accessed on date July 2020]

5 NHS. HPV Vaccine Overview. 2017. Available online: <https://www.nhs.uk/conditions/vaccinations/hpv-human-papillomavirus-vaccine/> [accessed on date April 2020]

6 NHS Inform. HPV Vaccine for Men Who Have Sex With Men (MSM). 2018. Available online: <https://www.nhsinform.scot/healthy-living/immunisation/vaccines/hpv-vaccine-for-men-who-have-sex-with-men-msm> [accessed on date April 2020]

7 NHS. HPV Vaccine Side Effects. 2017. Available online: <https://www.nhs.uk/conditions/vaccinations/hpv-vaccine-cervarix-gardasil-side-effects/> [accessed on date April 2020]

8 NICE. Human Papillomavirus Vaccines: Cautions. 2019. Available online: <https://bnf.nice.org.uk/drug/human-papillomavirus-vaccines.html#cautions> [accessed on date April 2020]

**Table S2.** Information on how variables were recoded.

| **Variable(s)** | **Original response options** | **Recoded response options** |
| --- | --- | --- |
| Awareness of HPV (1); | Yes | Heard of HPV |
| Awareness of vaccine | No; | Not heard of HPV |
|  | I don’t know |  |
| Awareness of HPV (2); | None at all; | Little/none |
| Awareness of anal cancer; | A little |  |
| Perceived severity of anal cancer; | A moderate amount; | Some |
| Anticipated regret | Quite a lot |  |
| Perceived severity of HPV | I don’t know | I don’t know |
|  | Not at all serious; | Little/none |
|  | Somewhat serious |  |
|  | Very serious; | High |
|  | Extremely serious |  |
| Female-only belief; | Strongly agree; | Variable present; e.g. female only belief, willing to disclose |
| Perceived benefits; | Somewhat agree |  |
| Self-efficacy; | Neither agree nor disagree | Neutral |
| Subjective norms; | Somewhat disagree; | Variable not present; e.g. no perceived benefits; no self-efficacy |
| Willingness to disclose; | Strongly disagree |  |
| Perceived behavioural control; |  |  |
| Fear of side effects (short term); |  |  |
| Fear of side effects (long term); |  |  |
| Logistical barriers; |  |  |
| Medication interaction; |  |  |
| Need for multiple shots |  |  |
| Perceived risk of HPV | I don’t know | I don’t know |
|  | Very unlikely; | Low |
|  | Unlikely |  |
|  | Neither likely nor unlikely | Neutral |
|  | Likely; | High |
|  | Very likely |  |
| Attitude to vaccine | Mostly positive; | Positive |
|  | Somewhat positive |  |
|  | Neutral / no opinion | Neutral |
|  | Somewhat negative; | Negative |
|  | Mostly negative |  |
| Perceived effectiveness (HPV); | I don't know | I don’t know |
| Perceived effectiveness (anal cancer) | No protection; | Little/none |
|  | A little protection; |  |
|  | A moderate amount of protection |  |
|  | Quite a lot of protection; | High |
|  | Complete protection |  |
| Ethnicity | Options containing “White” | White |
|  | Prefer not to say; | Other/prefer not to say |
|  | Other (please write) |  |
|  | Remaining options | BAME |
| Education | Primary school; | Below university |
|  | Secondary school; |  |
|  | Trade/technical school |  |
|  | Bachelor’s degree; | University and above |
|  | Post-graduate study |  |
| Hepatitis B vaccination | 0; | Did not initiate series |
|  | I don’t know if I have had any shots |  |
|  | 1; | Initiated series |
|  | 2; |  |
|  | At least one shot, but don’t know how many |  |
|  | 3 | Completed series |
| Number of sexual partners | [Open-ended response] | 0 partners |
|  |  | 1-12 partners |
|  |  | 13-50 partners |
|  |  | 51+ partners |
